# Optimal blood pressure control for patients after thoracic endovascular aortic repair of type B aortic dissection

**DOI:** 10.1186/s12872-019-1107-2

**Published:** 2019-05-27

**Authors:** Nan Lu, Xiaojing Ma, Tan Xu, Zhuoqiao He, Bayi Xu, Qingfeng Xiong, Xuerui Tan

**Affiliations:** 1 Department of Cardiology, the First Affiliated Hospital of Shantou University Medical College, No. 57, Changping Road, Shantou, Guangdong 515041 People’s Republic of China; 2 Image Center, Wuhan Asia Heart Hospital, Wuhan, Hubei 430000 People’s Republic of China

**Keywords:** Type B aortic dissection, Thoracic endovascular aortic repair, Optimal blood pressure, Aortic related adverse events

## Abstract

**Background:**

Guidelines recommend tight systolic blood pressure (SBP) control for favorable outcomes of type B aortic dissection (BAD) but are still limited by the optimal cut-off value of SBP. The purpose of this study was to evaluate the optimal cut-off value of SBP in BAD patients after thoracic endovascular aortic repair (TEVAR).

**Methods:**

From January 2011 to April 2017, 269 consecutive patients with BAD after TEVAR were included in the study. All patients were followed up according to a strict follow-up protocol. Cox regression analysis was used to examine the association between SBP at discharge and 90-day aortic related adverse events (ARAE).

**Results:**

All 269 patients completed 90 days of follow-up, and the unadjusted ARAE-free rates at 90-day was 95.1 ± 1.3%. The cut-off value of SBP at discharge identified by receiver operator curve was 130 mmHg for 90-day ARAE. In multivariable models, binary SBP at discharge was significant associated with 90-day ARAE (HR 3.780; 95% CI 1.236–11.556; *p* = 0.020). Hybrid operation (OR 2.046; 95%CI 1.015–4.122; *p* = 0.045) and insertion of ≥2 stents (OR 2.950; 95%CI 1.172–7.426; *p* = 0.022) were demonstrated to be independently associated with poor SBP control (SBP > 130 mmHg) using Logistic analysis.

**Conclusions:**

The optimal cut-off value of SBP at discharge was 130 mmHg which can be used to predict short-term ARAE. Blood pressure in patients with hybrid operation and ≥ 2 stents should be given more focus.

## Background

Stanford type B aortic dissection (BAD) is a life-threatening disease with high in-hospital and long-term follow-up mortality [1, 2]. Guidelines recommended tight blood pressure control for favorable outcomes but was still limited by certain cut-off level of systolic blood pressure (SBP), which was 120 mmHg, 130 mmHg, 135 mmHg or 140 mmHg [3–7]. For example, the newest European Society of Cardiology (ESC) guideline on the diagnosis and treatment of aortic diseases recommended that SBP should be maintained below 140 mmHg in chronic conditions in the “Treatment options” section, whereas maintenance below 130 mmHg in the “Long-term follow-up of aortic diseases” section was recommended [3]. Hypertension is a very important risk factor for aortic dissection. In principle, lower blood pressure is beneficial for patients with aortic dissection. However, too low blood pressure can cause signs or symptoms of ischaemia and complications of malperfusion, which would lead to failure or infarction of vital organs. In 2017, the American College of Cardiology (ACC) and American Heart Association (AHA) guideline for the management of high blood pressure in adults summarized that trials in patients with primary hypertension did not provide insight into the optimal blood pressure in patients with BAD [6]. Accordingly, evaluating the optimal cut-off value of SBP in BAD patients is very crucial and urgent.

Medical therapy, open surgery and thoracic endovascular aortic repair (TEVAR) therapy are treatment strategies for BAD [3]. TEVAR is associated with better survival compared to medical therapy or open surgery [3]. It is increasingly becoming the first-line treatment for BAD patients [8, 9]. Studies on blood pressure control of BAD were mostly after single medical therapy, rather than after TEVAR [3]. The International Registry of Acute Aortic Dissection (IRAD) study demonstrated that mortality after medical therapy was significantly increased in patients with BAD with refractory hypertension comparable to patients without refractory hypertension (35.6% versus 1.5%; *p* = 0.0003) [10]. However, there was no significant difference after TEVAR therapy between patients with and without refractory hypertension (3.7% versus 9.1%; *p* = 0.50) [10]. Those results may suggest that patients with BAD after TEVAR are less likely to need strict requirement of tight blood pressure control like the patients after medical therapy.

We performed a study to define the optimal SBP value for Chinese BAD patients after TEVAR. The optimal cut-off value of SBP was verified by aortic related adverse events (ARAE) and then risk factors for poor SBP control were assessed.

## Methods

### Study population

A retrospective study was conducted on prospectively collected data of BAD patients undergoing TEVAR from January 2011 to April 2017 at Wuhan Asia Heart Hospital (Wuhan, China). Patients with medical history of aortic diseases, Marfan syndrome or other connective tissue diseases, bicuspid aortic valve, iatrogenic or traumatic dissection, syphilis and other inflammatory diseases of the aorta, cancer, pregnancy, severe renal or respiratory or cardiac insufficiency were excluded. Patients who experienced acute myocardial infarction, cerebrovascular disease (CVD) or major surgical procedures before or after 30 days of study enrolment were also excluded.

Data on demographic characteristics, medical history, presenting symptoms, biochemical and imaging findings, treatment strategies, medication use, blood pressure at discharge, in-hospital outcomes and follow-up outcomes were collected. Discharge blood pressure was the last recorded blood pressure obtained within 24 h before or at hospital discharge.

The study was performed in accordance with the ethical standards laid down in the 1964 Declaration of Helsinki and its later amendments. All procedures were approved by the Ethics Committee of Wuhan Asia Heart Hospital. Written informed consent were obtained from all participants.

### Treatment and follow-up

All patients were treated with TEVAR and optimal antihypertensive medications. Each of the patients was measured by contrast-enhanced computed tomography (CT) before TEVAR procedure. TEVAR was performed according to the procedure described by Dake et al. [11] Successful TEVAR procedure was defined as technically successful placement of the stent graft at the intended target location without an endoleak [12]. Intravenous beta blockers and calcium channel blockers (CCB) were administered alone or combination to reduce SBP to < 120 mmHg as initial therapy in the intensive care unit. Intravenous medications were replaced by oral antihypertensive drugs including beta blockers, angiotensin-converting enzyme inhibitors (ACEI), angiotensin receptor blockers (ARB), CCB and diuretics, either alone or in combination. The decision when, whether and how to give oral antihypertensive medications was at the discretion of the treating physician according to current guidelines and the best clinical practice. Hypertension was defined as a self-reported diagnosis of hypertension, treatment with antihypertensive agents or having a clinical record of a blood pressure ≥ 140/90 mmHg^6^. All subjects were classified into the acute group (≤ 14 days), sub-acute group (14–90 days) and the chronic group (> 90 days), based on the time interval from the symptom onset date to the procedure date [3]. Body mass index (BMI) is equal to weight (kg) divided by the square of height (m^2^). Overweight was defined as a BMI between 25 kg/m^2^ and 30 kg/m^2^ and obesity as a BMI ≥30 kg/m^2^ [13].

All patients were followed up by 3 clinical cardiologists. Data on symptoms, medications, laboratory measurements, electrocardiograms, echocardiograms and contrast-enhanced CT findings were collected through electronic data capture and telephone interviews. Thirty days and 90 days after the completion of TEVAR were designed to follow up. Ninety-day ARAE was used for statistical analysis in our study.

ARAE were defined as aortic related death, new dissection, progression of aortic dissection (aortic rupture, necessitating surgical procedure or TEVAR after discharge), malperfusion (bowel ischemia, renal ischemia and lower limb ischemia), paraplegia, major stroke or endoleaks [14–17].

### Statistical analysis

One-Sample Kolmogorov-Smirnov test was used to evaluate the distribution of all variables. Age was a normal continuous variable and was presented as means ± standard deviations. SBP, DBP, pulse pressureand heart rate at discharge were non-normal continuous variables and were presented as medians (quartile 1 to quartile 3 [Q1–Q3]). Sex, the classification of BMI, tobacco abuse, alcohol abuse, hypertension, DM, PAD, CVD, CAD, dyslipidemia, stage of BAD, operative procedure, number of stentand medications at discharge were categorical variables and they were shown as counts and percentages. Independent samples *t*-test, Mann-Whitney U test and Chi-square test were used to compare the differences betweennormal continuous variables, non-normal continuous variables and categorical variables, respectively. SBP as a continuous variable predictor of the endpoint of 90-day ARAE was analyzed by receiver operator characteristic (ROC) curves. The optimal cut-off points of SBP to differentiate ARAE from non-ARAE outcomes were determined using Youden’s index, which equally weighs sensitivity and specificity and maximizes the number of correctly classified patients. Overall ARAE-free probabilities were estimated using the Kaplan-Meier method and comparisons between groups were carried out using the log-rank test. The relationship between SBP at discharge and 90-day ARAE was assessed using univariate and multiple cox proportional hazard analysis. Logistic analysis was performed to identify independent risk factors for poor blood pressure control. Hazard rates (HR) and odds ratios (OR) were assessed with a 95% confidence interval (CI), and a two-tailed *p* < 0.05 was considered statistically significant. All data analyses were performed using the statistical software Statistical Product and Service Solution (SPSS 19.0 for windows, Chicago, Illinois, USA).

## Results

### Demographics

There were 292 consecutive BAD patients who were initially treated with TEVAR in Wuhan Asia Heart Hospital. Among them, 1 patients who presented with other aortic lesions besides BAD, 1 with medical history of aortic diseases, 1 with Marfan syndrome, 1 with syphilitic aortic disease, 3 with iatrogenic or traumatic dissection, 5 with cancer, 3 with renal insufficiency, 2 patients who had CVD within 30 days of study enrolment, 4 patients who underwent surgical procedures within 30 days of study enrolment and 2 patients without follow-up records were excluded. Finally, 269 patients were enrolled.

All 269 patients completed 90 days of follow-up, and the unadjusted ARAE-free rates at 90-day was 95.1 ± 1.3%. The mean age of all patients was 55 ± 10 years, and 84.0% of the patients were males. Patient demographics, clinical characteristics, treatment, blood pressure and medications at discharge are outlined in Table 1.

**Table 1:** Baseline characteristics of patients with type B aortic dissection after TEVAR

	Overall *n* = 269	SBP ≤ 130 mmHg group *n* = 229	SBP > 130 mmHg group *n* = 40	*p*
Males, n (%)	226 (84.0)	190 (83.0)	36 (90.0)	0.263
Age (year)	55.5 ± 9.9	55.5 ± 9.9	55.9 ± 9.8	0.818
BMI, n (%)				0.048
Normal	148 (55.0)	127 (55.5)	21 (52.5)	
Overweight	108 (40.2)	94 (41.0)	14 (35.0)	
Obesity	13 (4.8)	8 (3.5)	5 (12.5)	
Tobacco abuse, n (%)	177 (65.8)	150 (65.5)	27 (67.5)	0.806
Alcohol abuse, n (%)	62 (23.0)	52 (22.7)	10 (25.0)	0.751
Hypertension, n (%)	240 (89.2)	203 (88.6)	37 (92.5)	0.468
DM, n (%)	26 (9.7)	22 (9.6)	4 (10.0)	0.938
PAD, n (%)	124 (46.1)	105 (45.9)	19 (47.5)	0.847
CVD, n (%)	100 (37.2)	79 (34.5)	21 (52.5)	0.030
CAD, n (%)	54 (20.1)	42 (18.3)	12 (30.0)	0.089
Dyslipidemia, n (%)	190 (70.6)	159 (69.4)	31 (77.5)	0.301
Stage of BAD, n (%)				0.020
Acute	163 (60.6)	143 (62.5)	20 (50.0)	
Sub-acute	60 (22.3)	53 (23.1)	7 (17.5)	
Chronic	46 (17.1)	33 (14.4)	13 (32.5)	
Operative procedure, n (%)				0.041
TEVAR	191 (71.0)	168 (73.4)	23 (57.5)	
Hybrid operation	78 (29.0)	61 (26.6)	17 (42.5)	
Number of stent, n (%)				0.017
1	243 (90.3)	211 (92.1)	32 (80.0)	
≥ 2	26 (9.7)	18 (7.9)	8 (20.0)	
SBP at discharge, mm Hg	120 (115–130)	120 (114–125)	140 (135–140)	< 0.001
DBP at discharge, mm Hg	70 (68–75)	70 (68–70)	76 (70–80)	< 0.001
Pulse pressure at discharge, mm Hg	50 (64–72)	50 (42–54)	61 (58–70)	< 0.001
Heart rate at discharge, beats per minute	68 (64–72)	68 (64–72)	70 (63–74)	0.255
Medications at discharge, n (%)				
ACEI/ARB	242 (90.0)	205 (89.5)	37 (92.5)	0.563
CCB	240 (89.2)	205 (89.5)	35 (87.5)	0.704
Beta blocker	260 (96.7)	220 (96.1)	40 (100.0)	0.202
Diuretic	72 (26.8)	59 (25.8)	13 (32.5)	0.375
Statin	206 (76.6)	177 (77.3)	29 (72.5)	0.509

*BMI* body mass index, *DM* diabetes mellitus, *PAD* peripheral arterial disease, *CVD* cerebrovascular diseases, *CAD* coronary artery disease, *BAD* type B aortic dissection, *GFR* glomerular filtration rate, *TEVAR* thoracic endovascular aortic repair, *SBP* systolic blood pressure, *DBP* diastolic blood pressure, *ACEI* angiotensin-converting enzyme inhibitors, *ARB* angiotensin receptor blockers, *CCB* calcium-channel blockers

### Selecting cut-off values of SBP to discriminate ARAE

We constructed ROC curves of the SBP for the diagnosis of 90-day follow-up ARAE to find the optimal cut-off value with the highest Youden index. The optimal cut-off value of SBP at discharge for 90-day ARAE was 131 ≈ 130 mmHg. Subsequently, all the patients were divided into SBP ≤ 130 mmHg group and SBP > 130 mmHg group when the 90-day outcomes were analysed.

### Risk factors forARAE

**Fig. 1:**
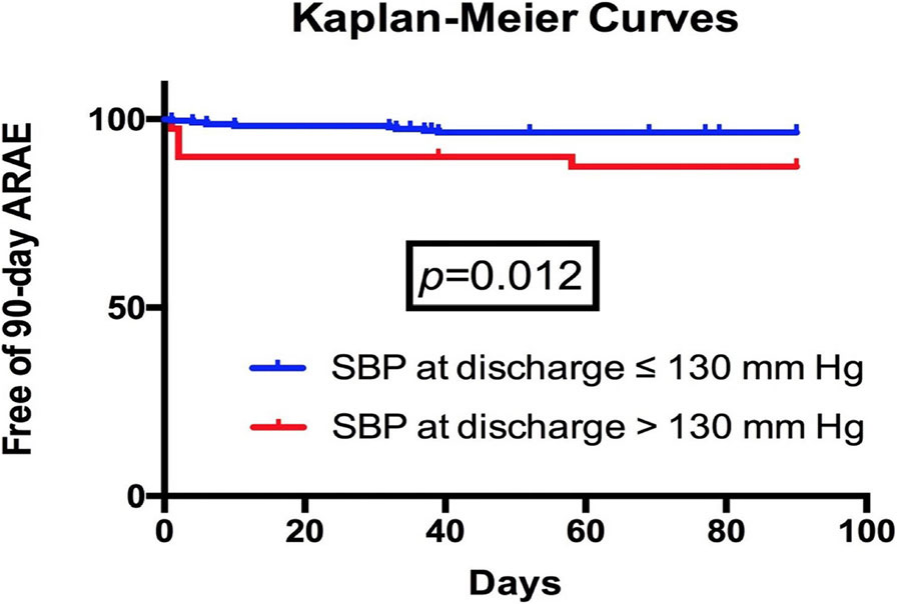
Free of ARAE of patients divided by systolic blood pressure at discharge. Kaplan-Meier calculation of freedom from development of ARAE in BAD patients after TEVAR by grouping with systolic blood pressure at discharge. (ARAE: aortic-related adverse events; BAD: type B aortic dissection; TEVAR: thoracic endovascular aortic repair)

Cumulative ARAE-free rate in all patients was 95.1 ± 1.3% at 90-day follow-up. The probability of 90-day ARAE-free as categorized by SBP using Kaplan-Meier analysis showed significant differences among the two groups (SBP ≤ 130 mmHg group vs. SBP > 130 mmHg group, *p* = 0.012), as shown in Fig. 1. Multivariate Cox analysis was performed. SBP at discharge (HR 3.780; 95% CI 1.236–11.556; *p* = 0.020) was the only significant predictor of 90-dayARAE.

### Risk factors for poor SBP control

All patients were divided into 2 groups according to the significant cut-off value of SBP at discharge: SBP ≤ 130 mmHg group and SBP > 130 mmHg group. Table 1 shows the details of patient characteristics categorized by SBP group. BMI, CVD, stage of BAD, operative procedures and number of stents were significantly different between the two groups. Hybrid operation (OR 2.046; 95%CI 1.015–4.122; *p* = 0.045) and insertion of ≥2 stents (OR 2.950; 95%CI 1.172–7.426; *p* = 0.022) were demonstrated to be independently associated with poor SBP control (SBP > 130 mmHg) by Logistic analysis (Table 2).

**Table 2:** Multiple logistic analysis of risk factors for poor blood pressure control

	OR	95% CI	*p* value
Operative procedure, n (%)			
TEVAR	Reference		
Hybrid operation	2.046	1.015–4.122	0.045
Number of stent, n (%)			
1	Reference		
≥ 2	2.950	1.172–7.426	0.022

*TEVAR* thoracic endovascular aortic repair

## Discussion

In the present, we examined the optimal cut-off value of SBP at discharge and the risk factors for poor blood pressure control. The main findings showed that lowering SBP at discharge to a target goal of ≤130 mmHg, compared with> 130 mmHg, resulted in significantly lower rates of 90-day ARAE. Logistic analysis demonstrated that significant predictors of poor blood pressure include hybrid operations and insertion of ≥2 stents.

The results presented herein are consistent with hemodynamic mechanismsin patients with BAD to stabilize the aortic wall and prevent ARAE [3]. A much higher blood pressure may further increase the risk of ARAE as Delsartet al. demonstrated that an SBP more than 130 mmHg was associated with more aortic enlargement, which has already been described as a risk factor of ARAE in patients with BAD [18]. While significantly lowering blood pressure can possibly compromise organ perfusion, and increase 30-day mortality in patients with ruptured abdominal aortic aneurysm, particularly in the older patient with other cardiovascular diseases and high cardiovascular resistance [19].

The blood pressure target for patients with type A aortic dissection and BAD should be different. Type A dissections have the worst prognosis with an overall in-hospital mortality of 30%, whereas BAD tends to have a better prognosis than type A dissections, having an overall in-hospital mortality rate of 13% [20]. A recent study assessed the effect of blood pressure on 260-month outcomes following repair of type A acute dissection [21]. Patients with a SBP < 120 mmHg had a reduced risk of reoperation compared with those having a SBP 120–140 mmHg or > 140 mmHg by Kaplan-Meier analysis. In our study, 130 mmHg was demonstrated to be the target SBP for BAD patients. This value is slightly higher than 120 mmHg, which was derived from the above study of type A aortic dissection patients. Therefore, the blood pressure target of 130 mmHg is reasonable for BAD patients. However, the detailed difference of blood pressure control between type A aortic dissection and BAD need to be investigated in a future study.

In general, different treatments, including medical, TEVAR and surgery, are suitable for BAD patients with different serious conditions. For BAD patients with different therapies, the standard of decrease of blood pressure possibly shall vary. Theoretically, the cut-off value of SBP is more likely to need a lower level in BAD patients treated with simple medical than that after TEVAR, as the aortas of the latter are protected by stents. After the specific treatment plan, the level of blood pressure control should be regulated for BAD patients treated with variable surgery. In brief, multi-center, large sample studies are needed to clarify these problems in the future.

In this study, we recorded blood pressure at discharge instead of at follow-up. We consider that blood pressure at discharge is inconsistence with the one recorded at follow-up, indicating different implication. Blood pressure at follow-up indicates the management of blood pressure among a period of time after discharge, while the one at discharge demonstrates a better state of blood pressure in BAD patients after regular management by clinicians in inpatient department. Recent years, several studies have confirmed that SBP at discharge was a significant predictor for long-term outcomes [22, 23]. Even though, further studies are needed to determine the association between blood pressure at discharge and long-term follow-up and to define an optimal long-term follow-up blood pressure range for BAD patients after TEVAR.

CVD was a risk factor of ARAE in BAD patients after TEVAR in the present study. Based on previous studies, CVD patients were older, more often had hypertension and atherosclerosis, and presented more frequently with symptoms such as syncope, hypotension, shock and pulse deficit [24]. These factors were all associated with an increased prevalence of morbidity and other complications in BAD patients [3]. Therefore, aortic dissection patients with CVD may have a high predicted mortality, and this association was also demonstrated in a previous study [25].

Hybrid operation and insertion of ≥2 stents were risk factors for poor blood pressure. Hybrid operations were performed in patients involving the distal aortic arch. BAD involving the distal aortic arch are characterized by a high risk of rapidly expanding false lumens which may result in increments of vascular resistance and then effect blood pressure management. Patients with ≥2 stents presented with a large range of dissection, which mainly involved renal arteries, as described by a previous study [26]. A study further demonstrated that patients with renal artery dissections presented with severe hypertension [27]. Consequently, patients with ≥2 stents may have a greater risk of high pressure blood.

Antihypertensive medications play a main role in the management of BAD. Current practice in the treatment of chronic BAD is the use of beta-blockers as first-line therapy to reduce the force of left ventricular ejection, decrease aortic wall stress and improve survival [6]. ESC 2014 guidelines on the diagnosis and treatment of aortic dissection regarded beta-blockers as initial antihypertensive medications [3]. However, our data failed to show the benefit of the routine use of beta-blockers. Because beta-blockers were prescribed to 96.7% of patients in our study and the statistical analysis were limited to demonstrated significant results. Other antihypertensive medications, such as CCB, ACEI and ARB have been suggested for the medical therapy of BAD. However, these suggested antihypertensive medications did not improve survival in a series of patients with type A and type B aortic dissections [28]. Our results are consistent with these previous findings.

### Limitations

There are several limitations to the present study. First, the monitor of ambulatory blood pressure and blood pressure variability providng insight into overall blood pressure control for outpatient during follow-up were not performed. Second, the present study is a single-center study and multi-center studies in type BAD are required in the future. Finally, the choice of antihypertensive medications was leftat the physician’s discretion and subject to potential selection bias.

## Conclusions

The optimal cut-off value of SBP at discharge was 130 mmHg which can be used to predict 90-day ARAE. It may be difficult to control SBP at discharge for patients with hybrid operation and ≥ 2 stents. Further studies on blood pressure might provide new preventive and therapeutic strategies for aortic dissection.

## Data Availability

Raw data supporting the obtained results are available at the corresponding author.

## Abbreviations

ACC: The American college of cardiology
ACEI: Angiotensin-converting enzyme inhibitors
AHA: The American heart association
ARAE: Aortic related adverse events
ARB: Angiotensin receptor blockers
BAD: Stanford type B aortic dissection
BMI: Body mass index
CCB: Calcium channel blockers
CI: Confidence interval
CT: Computed tomography
CVD: Cerebrovascular disease
ESC: European society of cardiology
HR: Hazard rates
IRAD: The international registry of acute aortic dissection
OR: Odds ratios
ROC: Receiver operator characteristic
SBP: Systolic blood pressure
TEVAR: Thoracic endovascular aortic repair
